# Parkinson disease in Gaucher disease

**DOI:** 10.1186/s40734-017-0054-2

**Published:** 2017-05-23

**Authors:** Federico Rodriguez-Porcel, Alberto J. Espay, Miryam Carecchio

**Affiliations:** 1 James J. and Joan A. Gardner Center for Parkinson disease and Movement Disorders, Department of Neurology and Rehabilitation Medicine, University of Cincinnati, 260 Stetson St., Suite 2300, Cincinnati, OH 45267-0525 USA; 2 Molecular Neurogenetics Unit, IRCCS Foundation Carlo Besta Neurological Institute, Milan, Italy; 3 Department of Pediatric Neurology, IRCCS Foundation Carlo Besta Neurological Institute, Milan, Italy; 4 Department of Molecular and Translational Medicine, University of Milan-Bicocca, Milan, Italy

**Keywords:** Parkinson disease, Gaucher disease, Glucocerebrosidase

## Abstract

**Background:**

Gaucher disease (GD) is an inborn error of metabolism caused by mutations in the gene (*GBA*) coding for glucocerebrosidase (GCase), inherited in an autosomal recessive pattern. GD patients have up to 9% risk of developing PD.

**Case presentation:**

We report two patients with GD that developed PD at different disease stages.

**Conclusion:**

We reviewed the literature on the coexistence of PD and GD and speculate that the severity of symptoms may be related to the type of GBA mutation inherited.

**Electronic supplementary material:**

The online version of this article (doi:10.1186/s40734-017-0054-2) contains supplementary material, which is available to authorized users.

## Background

Gaucher disease (GD) is an autosomal recessive inborn error of metabolism caused by mutations in the gene (*GBA*) coding for the enzyme glucocerebrosidase (GCase). Abnormal ß-glucocerebrosidase function results in the accumulation of glucosylceramide in lysosomes of reticuloendothelial cells, leading to a variety of systemic manifestations, including organomegaly, anemia, thrombocytopenia, and bone disease. The associated systemic inflammatory response plays a major role in the pathophysiology of GD [1].

Depending on clinical features and progression, GD has been traditionally classified into three clinical subtypes. The most common subtype, type 1, has been classically distinguished from the others by the lack of central nervous system manifestations [1]. GD with neurologic involvement (neuronopathic GD) usually appears in childhood and is classified as type 2 or 3 based on the rapid or slowly progressive course of neurologic symptoms, respectively. However, half of those with GD type 1 (GD1) present neurological symptoms and 30% of GD1 patients without neurological complaints show abnormalities on examination if carefully evaluated [2, 3]. For these reasons, and taking into consideration the extremely variable age at onset and complexity of manifestations, the traditional classification of GD has come under scrutiny given a disease spectrum of variable severity.

Parkinson disease (PD) is present in 4% of GD1 patients [2]. Even when PD criteria are not met, over 21% of GD1 patients may exhibit at least one parkinsonian finding [2]. In this report, we present two patients with GD who developed parkinsonism at different disease stages.

## Case Presentation

### Case 1

This 42-year-old man complained of generalized stiffness since he was a child, which worsened in the year prior to his evaluation. Right-hand tremor and slow gait had become prominent. He endorsed poor balance but no falls. His main source of disability was a cramp-like pain in his arms and legs. He had tried gabapentin, amitriptyline, topiramate, and duloxetine without any benefits. He acknowledged anosmia and constipation.

He received a diagnosis of GD1 (N370S homozygous) when he was 18 months old after evaluation for splenomegaly. He was born to non-consanguineous parents of Ashkenazi origin. Since then he had been on enzyme replacement therapy (ERT) with intravenous recombinant GCase (Velaglucerase alfa). He also had diabetes and small fiber peripheral neuropathy.

**Fig. 1:**
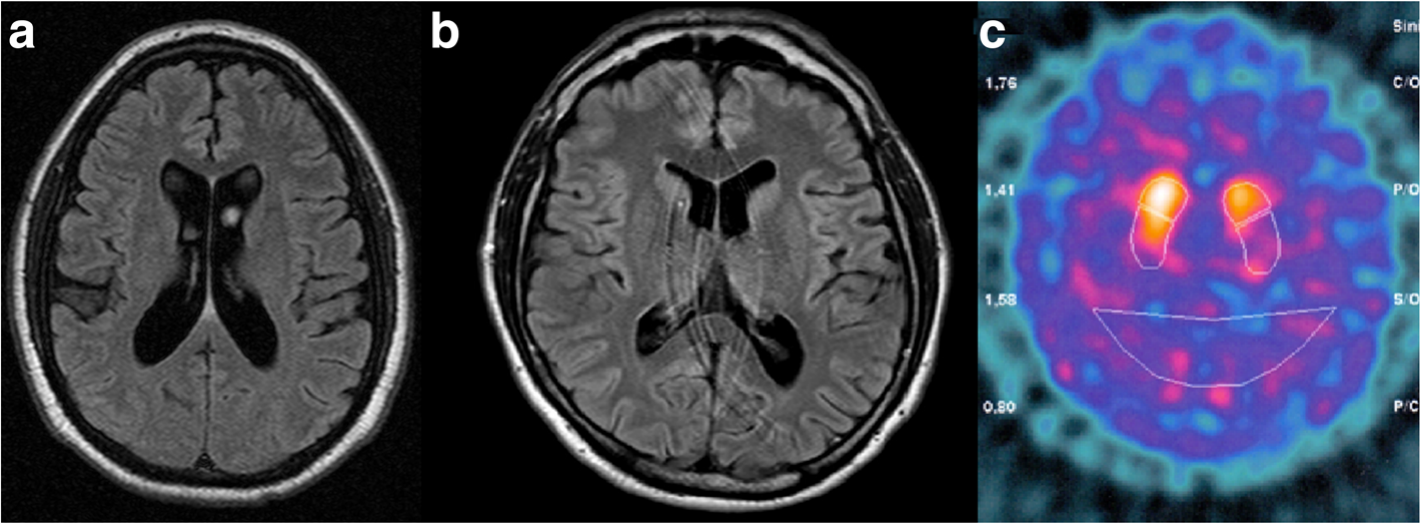
**a** FLAIR axial brain MRI exhibiting subtle cortical atrophy; (**b**) FLAIR axial brain MRI exhibiting mild cortical atrophy (**c**) Dopamine transporter scan (DaTScan) exhibiting bilateral decrease in tracer uptake, greater in the left putamen and, to a lesser extent, caudate nuclei



On examination, he had marked splenomegaly. He exhibited asymmetric bradykinesia and rigidity, with mild left leg spasticity and postural impairment. There was a mild restriction of horizontal gaze and slowness of saccades, both present when looking to the right (Additional file 1: Video S1). He had symmetric distal hypoesthesia to all sensory modalities up to knees and wrists. The motor subscale of the Movement Disorder Society-Unified Parkinson’s Disease Rating Scale (MDS-UPDRS-III) score was 17. Montreal Cognitive Assessment score was 27/30. Glutamic acid decarboxylase antibody was negative. Brain MRI showed subtle diffuse cortical atrophy (Fig. 1a).

**Additional file 1: Video S1.** Note the bradykinesia with sequential decrement, slow gait, mild restriction in horizontal gaze and saccadic slowing when looking to the right. (MP4 66235 kb)


He reported improvement in leg stiffness and pain with levodopa 100 mg three times daily, which was associated with partial improvement of bradykinesia and rigidity on examination, with a reduction (improvement) in the MDS-UPDRS-III score to 11.

### Case 2

This 48-year-old man had a 3-year history of progressive generalized slowness. His wife noted social withdrawal and apathy within the same period. He had also developed insomnia. His mother died at the age of 72 years from a hepatic malignancy and was suspected to have mild cognitive impairment. No additional family members had a history of neurological or psychiatric disease.

On examination, he had a palpable splenomegaly. There was asymmetric bradykinesia and rigidity associated with hypomimia and hypophonia. Gait had a normal base and stride length, with reduced swing and flexed posture of the right arm (Additional file 2: Video S2). Complete blood count demonstrated mild thrombocytopenia (130,000/mm^3^). Brain MRI showed mild diffuse cortical atrophy (Fig. 1b). DAT-SPECT showed severe striatal tracer uptake reduction, more marked in the left caudate nucleus (Fig. 1c). There were no bone abnormalities reported.



**Additional file 2: Video S2.** Note the decreased blink rate, asymmetric slow rapid alternating movements more and absent arm swing on the right side with semi-flexion of the forearm. (MP4 32578 kb)


He was found to be compound heterozygous for *GBA* mutations (N370S/L444P). Segregation analysis was performed and his parents were found to be carriers of the mutations. He was started on enzyme replacement therapy with imiglucerase, in addition to escitalopram and ropinirole. Parkinsonian features were mildly improved with levodopa (titrated up to 450 mg/day) and ropinirole (to 20 mg/day). Higher doses of levodopa were not tolerated because of confusion and severe nausea, refractory to domperidone.

## Discussion

These two patients with GD1 had early-onset parkinsonism with partial benefit from dopaminergic therapy. In the first case, the diagnosis of GD1 had been made in early infancy while in the second in adulthood by the combination of parkinsonism, splenomegaly, and thrombocytopenia.

The relationship between PD and *GBA* has been unraveled in recent years. Patients with GD1 have a 4-9% risk of developing PD, a similar risk to that of *GBA* carriers [4]. The risk of developing PD in GD1 is higher in males and increases with age [5]. Systemic non-neurologic manifestations of GD usually precede parkinsonian symptoms and must always be investigated in the case of early-onset PD or atypical parkinsonian cases [5]. The clinical presentation of GD-PD can be indistinguishable from non-*GBA* and *GBA* carriers with the most common being an asymmetric resting tremor [6]. However, other associated features, including horizontal supranuclear gaze palsy, subcortical myoclonus, and pyramidal signs may present in GD-associated PD [7, 8]. GD-PD patients have a high prevalence of non-motor symptoms, most commonly anosmia and dysautonomia [7]. Cognitive impairment with a more rapid cognitive decline is common [7]. Mood and behavioral symptoms, particularly depression, may be found more frequently, as documented in the second case [7]. Benefit from levodopa is variable, with most reports describing partial and/or temporary benefit [9]. Early fluctuations and dyskinesia are also common in this group of patients [7]. Deep brain stimulation has been reported as beneficial in two patients [6].

Unlike our cases where subtle to mild diffuse atrophy was observed, brain MRI is usually reported as normal. Dopamine transporter imaging shows reduced tracer uptake [10]. In pathological studies, GD-PD patients demonstrate a wider spread of Lewy bodies, particularly in neuronal populations of the CA2-CA4 hippocampal subregions [8]. Cortical atrophy and Involvement of the hippocampus could explain the increased incidence of cognitive impairment.

Current treatments for GD include enzyme replacement therapy (ERT) and substrate-reduction therapies (SRT). ERT is based on the intravenous administration of recombinant GCase, which does not cross the blood-brain barrier [11]. SRT reduces the accumulation of glucosylceramide and can cross the blood-brain barrier. Neither treatment has been shown to improve the parkinsonism or any other neurological endpoint in GD-PD.

GD can present other neurological features besides parkinsonism, most commonly neuropathy, as seen in the first case [3]. The nature of the neuropathy is likely multifactorial given a higher incidence in GD of monoclonal gammopathy and diabetes, the latter in patients undergoing ERT [12]. Adults with GD1 have a higher risk of cerebrovascular disease; children can present with oculomotor apraxia, deafness or myoclonic epilepsy.

*GBA* mutations represent one of the many disorders affecting lysosomal function and increasing the risk for parkinsonism (Additional file 3: Table S1). However, mechanisms by which *GBA* mutations increase the risk for PD are not fully understood. Although management of PD in carriers or affected patients with *GBA* mutations does not differ from non-carriers, identification of a *GBA* mutation has implications for genetic counseling. While measurement of GCase activity is suggested, but not required, for the diagnosis of GD in patients with well-known *GBA* mutations, it is essential in order to establish the pathogenic nature of those carrying novel mutations where causality is not confirmed. Currently, it is not possible to ascertain which GD patients or *GBA* mutation carriers are at risk of developing PD. However, the varying GD spectrum may be dependent on the type of *GBA* mutations and their corresponding residual enzymatic activity [1]. “Null” or “severe” *GBA* mutations (e.g. L444P) are associated with onset during infancy and childhood, rapid progression, shorter life expectancy, and appearance of more severe neurologic features (GD2 and GD3). The “mild” GD1 mutations (e.g., N370S) yield more benign phenotypes without minimal or no neurologic deficits on presentation [1]. Half of the patients with GD-PD are homozygous for N370S and over 90% of GD-PD patients carry at least one N370S mutation [9]. However, there likely is an ascertainment bias given the high prevalence of N370S among Ashkenazi Jews, on which most studies have been based. Moreover, GD systemic symptoms are usually milder in those who develop PD [9]. We speculate that PD may represent a comparatively benign neurologic manifestation of GD1, of later onset, while carriers of “severe” mutations do not live to develop parkinsonism due to their shorter life spans with other, more malignant neurologic features. Interestingly, in GBA mutation carriers, “severe” mutations have a higher risk of PD than “mild” mutations (OR: 2.2 [CI 1.5–3.1] vs 13.6 [CI 7.2–25.9]) as well as an earlier onset of symptoms (55.7 vs 57.9 years) [13].

## Conclusion

Our cases illustrate early-onset PD in GD. The difference in the severity of the presentation may be accounted by their genotypes, with the first case being homozygous for a “mild” mutation, while the second carried a “severe” mutation. Other possible phenotypic modifiers include prolonged treatment exposure to ERT in the first case, affecting the severity of the symptoms by reducing systemic inflammation. Our cases also highlight the variable phenomenology and overlap between GD types and suggest further research will be needed to examine the diverse contributions of *GBA* mutations to biological and epigenetic factors associated with neurological disease.

## Consent

Both patients gave informed consent for the publication of their case, videos, and ancillary data. Data was de-identified.

## Additional files

Additional file 3: **Table S1.** Selected lysosomal disorders associated with parkinsonism. (DOCX 29 kb)

## Abbreviations

ERT: Enzyme replacement therapy
*GBA*: Glucocerebrosidase gene
GCase: Glucocerebrosidase enzyme
GD: Gaucher Disease
MDS-UPDRS: Movement Disorder Society-Unified Parkinson’s Disease Rating Scale
PD: Parkinson disease
SRT: Substrate-reduction therapies
