# Supplementary material for: Parkinson disease in Gaucher disease
Source: J Clin Mov Disord. 2017 May 23;4:7. doi: 10.1186/s40734-017-0054-2 (PMC5440911; doi:10.1186/s40734-017-0054-2)
Supplement: Supplementary file 3 — Table S1. Selected lysosomal disorders associated with parkinsonism. (DOCX 29 kb) [file 40734_2017_54_MOESM3_ESM.docx]

**TABLE S1: Selected lysosomal disorders associated with parkinsonism**

| Disorder | Onset (years) | Additional motor findings | Non-motor features | Gene affected |
| --- | --- | --- | --- | --- |
| Parkinsonism with benefit from levodopa* | | | | |
| Gaucher^1^ | 30-60 | Horizontal SGP, myoclonus, dystonia. Levodopa-induced dyskinesia. | Hepato- and splenomegaly, anemia, thrombocytopenia, fractures. | *GBA* |
| Neuronal ceroid lipofuscinoses^2,3^ | 10-30 | Freezing of gait, facial myoclonus. | Visual loss, epilepsy, dementia. | Multiple genes (e.g. *CLN6, PPT1, CLN3, DNAJC5*) |
| Kufor-Rakeb^4^ | 10-30 | Spasticity, SGP, oculogyric spasms, facial-faucial-finger myoclonus. | Visual hallucinations, dementia. | *ATP13A2* |
| Chediak-Higashi^5^ | 17-58 | Ataxia, dystonia, peripheral neuropathy. | Albinism, immunodeficiency, bleeding diathesis. | *LYST* |
| Parkinsonism associated with ataxia | | | | |
| GM2 gangliosidosis  (Adult-onset)^6^ | 15-45 | Peripheral neuropathy, motor neuron disease. | Psychosis. | *HEX-A* |
| Niemann-Pick type C^7^ | 15-30 | Vertical SGP, dystonia, chorea, dysarthria. | Psychosis, cognitive decline, hepato- and splenomegaly. | *NPC-1, NPC-2* |
| Parkinsonism associated with spasticity | | | | |
| GM2 gangliosidosis  (Juvenile-onset)^8^ | 5-15 | Rigidity, ataxia. | Dementia, seizures. | *HEX-A* |
| X-linked parkinsonism with spasticity^9,10^ | 15-45 | Hyperreflexia, levodopa-induced dystonia. | Only men are affected. | *ATP6AP2* |
| Parkinsonism associated with generalized dystonia | | | | |
| GM1 gangliosidosis^11^ | 5-20 | Dysarthria. | Short stature, kyphoscoliosis, cardiomyopathy. | *GLB-1* |

SGP: supranuclear gaze palsy. * Lewy bodies can be found on autopsy for each of these cases, supporting the importance of impaired lysosomal function in PD.

1. Lopez G, Kim J, Wiggs E, et al. Clinical course and prognosis in patients with Gaucher disease and parkinsonism. *Neurology Genetics* 2016; **2**(2): e57.

2. Nijssen PC, Brusse E, Leyten AC, Martin JJ, Teepen JL, Roos RA. Autosomal dominant adult neuronal ceroid lipofuscinosis: parkinsonism due to both striatal and nigral dysfunction. *Movement disorders : official journal of the Movement Disorder Society* 2002; **17**(3): 482-7.

3. Damasio J, Taipa R, Melo-Pires M, et al. Freezing of gait--first motor manifestation in late infantile variant neuronal ceroid lipofuscinosis. *Parkinsonism & related disorders* 2014; **20**(2): 243-4.

4. Park JS, Blair NF, Sue CM. The role of ATP13A2 in Parkinson's disease: Clinical phenotypes and molecular mechanisms. *Movement disorders : official journal of the Movement Disorder Society* 2015; **30**(6): 770-9.

5. Balint B, Bhatia KP. Parkinsonism and Other Movement Disorders Associated with Chediak-Higashi Syndrome: Case Report and Systematic Literature Review. *Movement Disorders Clinical Practice* 2015; **2**(1): 93-8.

6. Inzelberg R, Korczyn AD. Parkinsonism in adult-onset GM2 gangliosidosis. *Movement disorders : official journal of the Movement Disorder Society* 1994; **9**(3): 375-7.

7. Mengel E, Klunemann HH, Lourenco CM, et al. Niemann-Pick disease type C symptomatology: an expert-based clinical description. *Orphanet journal of rare diseases* 2013; **8**: 166.

8. Neudorfer O, Pastores GM, Zeng BJ, Gianutsos J, Zaroff CM, Kolodny EH. Late-onset Tay-Sachs disease: phenotypic characterization and genotypic correlations in 21 affected patients. *Genetics in medicine : official journal of the American College of Medical Genetics* 2005; **7**(2): 119-23.

9. Korvatska O, Strand NS, Berndt JD, et al. Altered splicing of ATP6AP2 causes X-linked parkinsonism with spasticity (XPDS). *Human molecular genetics* 2013; **22**(16): 3259-68.

10. Poorkaj P, Raskind WH, Leverenz JB, et al. A novel X-linked four-repeat tauopathy with Parkinsonism and spasticity. *Movement disorders : official journal of the Movement Disorder Society* 2010; **25**(10): 1409-17.

11. Roze E, Paschke E, Lopez N, et al. Dystonia and parkinsonism in GM1 type 3 gangliosidosis. *Movement disorders : official journal of the Movement Disorder Society* 2005; **20**(10): 1366-9.
